# Supplementary material for: Surface Kondo effect and non-trivial metallic state of the Kondo insulator YbB12
Source: Nat Commun. 2016 Aug 31;7:12690. doi: 10.1038/ncomms12690 (PMC5515356; doi:10.1038/ncomms12690)
Supplement: Supplementary Information — Supplementary Figures 1-3 [file ncomms12690-s1.pdf]

Supplementary figures 1, 2 and 3 show the ARPES intensity plots and momentum and energy distribution curves. Their peak positions corresponding with the Figure 2(b), 3, 4(a, b) in the main text, are also indicated by triangle markers on each spectra.

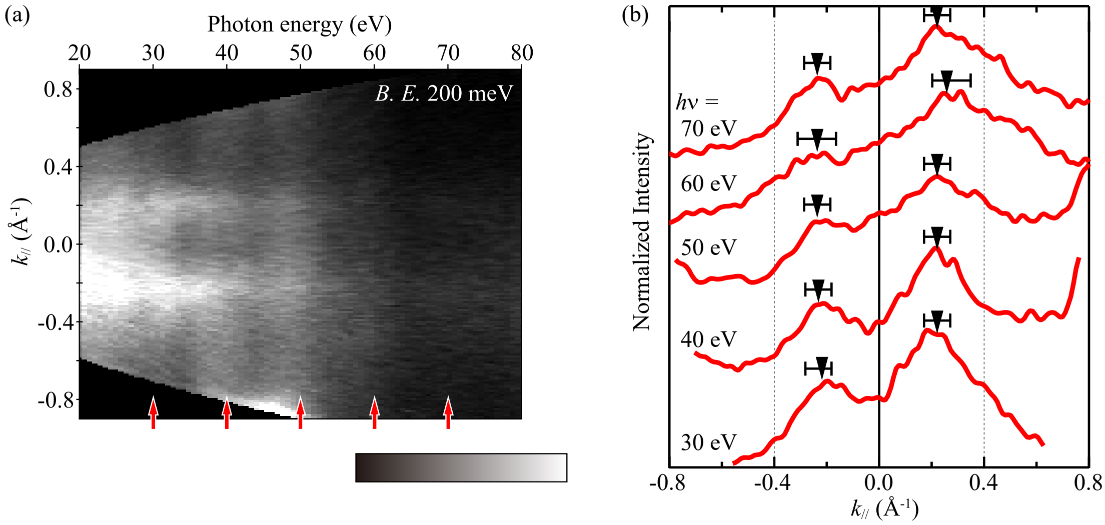

Supplementary Figure 1. **Detailed dataset of angle-resolved photoelectron spectroscopy (ARPES) photon-energy scan** (a) ARPES intensity plot along a dashed line in (a) at the binding energy of 200±10 meV as a function of photon energies taken at 20 K (The same data as Fig. 2 (b) in the main text). Arrows indicate the photon energies shown in (b). The intensity plots are shown in grayscale: white area represents high intensity. (b) Momentum distribution curves at photon energies from 30 to 70 eV.

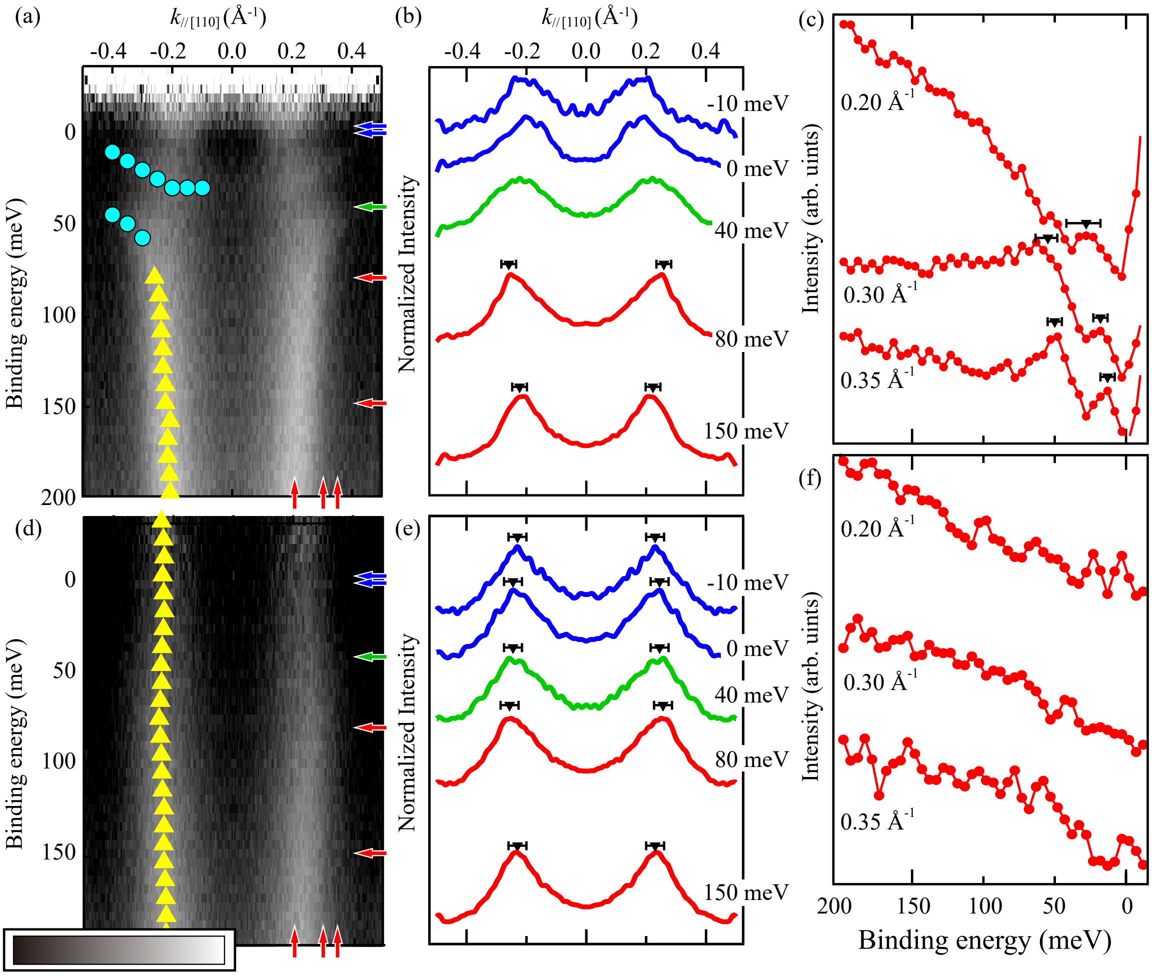

Supplementary Figure 2. **Detailed dataset of angle-resolved photoelectron spectroscopy (ARPES) measured with different temperatures** (a) ARPES intensity plot taken at 15 K divided by the Fermi distribution function convolved with the instrumental resolution with $h\nu$ = 16.5 eV (the same data as Fig. 3 (a) in the main text). The intensity plots are shown in grayscale: white area represents high intensity. (b) Momentum distribution curves and (c) energy distribution curves along the line indicated by the arrows in (a). (d-f) the same as (a-c) but taken at room temperature (300 K), respectively.

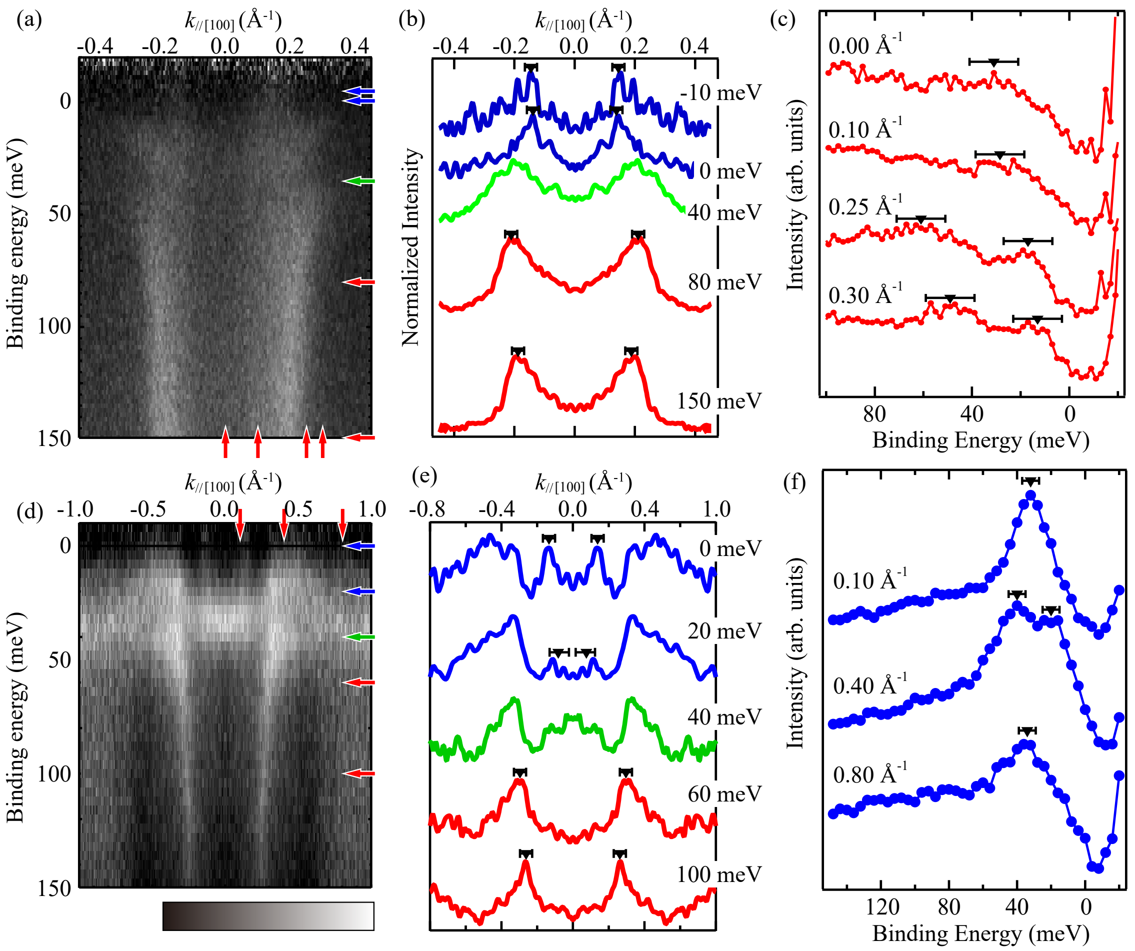

Supplementary Figure 3. **Detailed dataset of angle-resolved photoelectron spectroscopy (ARPES) showing topological surface states** (a) ARPES intensity plot taken at 20 K divided by the Fermi distribution function convolved with the instrumental resolution with $h\nu = 16.5$ eV (the same data as Fig. 4 (a) in the main text). The intensity plots are shown in grayscale: white area represents high intensity. (b) Momentum distribution curves and (c) energy distribution curves along the line indicated by the arrows in (a). (d-f) the same as (a-c), respectively, but taken at 14 K with $h\nu = 53.5$ eV.(the same data as Fig. 4 (b) in the main text).
